# Antiosteoporotic Effects of Huangqi Sanxian Decoction in Cultured Rat Osteoblasts by Proteomic Characterization of the Target and Mechanism

**DOI:** 10.1155/2015/514063

**Published:** 2015-10-18

**Authors:** Chong-Chong Guo, Li-Hua Zheng, Jian-Ying Fu, Jian-Hong Zhu, Yan-Xing Zhou, Tao Zeng, Zhi-Kun Zhou

**Affiliations:** ^1^Department of Pharmacy, Guangdong Medical College, No. 1, Xincheng Dadao, Songshan Lake Science and Technology Industry Park, Dongguan 523808, China; ^2^Department of Pharmacy, Sun Yat-Sen Memorial Hospital, Guangzhou 510120, China; ^3^Laboratory Medicine Center, Nanfang Hospital, Southern Medical University, Guangzhou, Guangdong 510515, China

## Abstract

Huangqi Sanxian decoction (HQSXD) is routinely used for the treatment of osteoporosis in the Chinese traditional healthcare system. However, the targets and mechanism underlying the effect of HQSXD on osteoporosis have not been documented. In the present study, seropharmacology and proteomic approaches (two-dimensional gel electrophoresis combined with mass spectrometry) were used to investigate the effects and possible target proteins of HQSXD on osteoblast. We found that HQSXD-treated rat serum significantly enhanced osteoblast proliferation, differentiation, and mineralization. In HQSXD-S-treated osteoblasts, there were increases in the expression of N-formyl peptide receptor 2 and heparan sulfate (glucosamine) 3-O-sulfotransferase 3A1 and reduction in the expression of alpha-spectrin, prohibitin, and transcription elongation factor B (SIII), polypeptide 1. The identified proteins are associated with cell proliferation, differentiation, signal transcription, and cell growth. These findings might provide valuable insights into the mechanism of antiosteoporotic effect affected by HQSXD treatment in osteoblasts.

## 1. Introduction

Osteoporosis is the most frequent bone remodeling disease and its incidence increases with advancing age. This disease is characterized by a reduction in bone mass and microarchitectural deterioration of bone tissue, resulting in high risk of fractures [1]. Globally, osteoporosis and associated bone fractures have become a major health hazard afflicting millions of people [2]. Current treatment options for osteoporosis include bisphosphonates, estrogens, selective estrogen receptor modulators, calcitonin, denosumab, and teriparatide. However, many of these drugs generate side effects [3, 4] and their costs are too high to benefit a large population in the developing and developed countries, which may limit their applications.

Traditional Chinese medicine has been a part of healthcare in China for thousands of years and has recently been reevaluated for clinical approach [5]. The traditional Chinese medicine has fewer adverse reactions and is more suitable for long-term administration than synthetic drugs and antibiotics. Popular commercially available prescriptions include Jinkui Shenqi Wan (JKSQW), which exerts a therapeutic effect on the kidney-Yang deficiency and osteoporosis indicated in Chinese Pharmacopoeia (2010), and Huangqi Sanxian decoction (HQSXD), a traditional Chinese formula, which is composed of* Radix Astragali*,* Epimedii Folium*,* Cistanche Herba*,* Radix notoginseng*,* Radix Salviae Miltiorrhizae*,* Corydalis Rhizoma*, Radix Angelicae Sinensis, and* Radix Clematidis*. Our previous study revealed that Huangqi Sanxian decoction treatment significantly increased sex estrogen level and bone mineral density (BMD) and repressed bone absorption function in postmenopausal women [6]. This suggested that HQSXD has beneficial effects in the treatment of osteoporosis. However, little is known about the mechanisms and targets underlying the effects of HQSXD on osteoporosis.

The Chinese traditional medicine theory believes that bone activities are controlled by the kidney. Strong “kidney” can nourish bones, but the weak “kidney” might hasten bone deterioration [7]. Kidney deficiency and blood stasis are the main pathological basis of osteoporosis. Huangqi Sanxian decoction is composed of eight Chinese medicinal herbs; of these,* Epimedii Folium* and* Cistanche Herba* strengthen kidneys, while* Radix Salviae Miltiorrhizae* and* Radix notoginseng* invigorate the circulation of blood. These herbs are an excellent combination for highlighting their superiority in the treatment of osteoporosis [8].

Jinkui Shenqi Wan, an ancient Chinese herbal formula, is indicated in the Chinese Pharmacopoeia (2010) for the treatment of Yang insufficiency of kidney, weakness and soreness of the loins and the knees, cold feeling in the limbs, and frequent urination. Human clinical studies have certified that Jinkui Shenqi Wan exerts a therapeutic effect on the kidney-Yang deficiency [9]. Hence, we used Jinkui Shenqi Wan as a positive control in this study.

In the present study, seropharmacology and functional proteomics technology were used to explore the multiple proteins associated with the antiosteoporotic effect. The results suggest a basis for the clinical use of HQSXD in the treatment of patients with osteoporosis.

## 2. Materials and Methods

### 2.1. Animals

Thirty Sprague-Dawley rats (fifteen male and fifteen female), weighing about 250–300 g, were purchased from the Animal Experimental Center of Guangdong Medical College (Dongguan, China). The animals were housed individually in a regulated environment (24 ± 0.5°C), with a 12-hour light/dark cycle (under light: 08:00–20:00 h). Food and water were given* ad libitum* throughout the experiment. After three days of acclimation, male and female SD rats were randomly divided into three groups: blank-control group, experimental group treated with Huangqi Sanxian decoction, and positive control group treated with Jinkui Shenqi Wan. The Committee of Experimental Animal Administration of the University approved the study, and the procedures of the experiment were in accordance with generally accepted international rules and regulations.

### 2.2. Preparation of HQSXD and JKSQW

HQSXD included eight plant extracts, including* Radix Astragali* (root, Chinese herbal name: Huang-Qi),* Epimedii Folium* (leaf, Chinese herbal name: Yin-Yang-Huo),* Cistanche Herba* (succulent stem, Chinese herbal name: Rou-Cong-Rong),* Radix notoginseng *(root and rhizome, Chinese herbal name: San-Qi),* Radix Salviae Miltiorrhizae *(root and rhizome, Chinese herbal name: Dan-Shen),* Corydalis Rhizoma* (rhizome, Chinese herbal name: Yan-Hu-Suo), Radix Angelicae Sinensis (root, Chinese herbal name: Dang-Gui), and* Radix Clematidis *(root and rhizome, Chinese herbal name: Wei-Ling-Xian) in a ratio of 15 : 10 : 10 : 5 : 10:10 : 8 : 10 [6]. The above eight medicinal extracts were obtained from Dongguan Sinopharm (Dongguan, China) and identified by Professor Zhou (Department of Pharmacy, Guangdong Medical College, Dongguan, China). The eight medicinal plant materials in the mixture (270 g) were powdered and coboiled with 1000 mL water for 2 hours. The extraction was repeated twice. The filtrates were concentrated to 200 mL under reduced pressure and kept at 4°C.

JKSQW consisted of* Radix Rehmanniae Preparata* (root, Chinese herbal name: Di-Huang),* Dioscoreae Rhizome* (root, Chinese herbal name: Shan-Yao),* Fructus Corni Officinalis* (fructus, Chinese herbal name: Shan-Zhu-Yu),* Sclerotium Poriae Cocos* (dried sclerotia, Chinese herbal name: Fu-Ling),* Cortex Moutan Radicis* (root bark, Chinese herbal name: Mu-Dan-Pi),* Rhizoma Alismatis Orientalis* (stem, Chinese herbal name: Ze-Xie),* Ramulus Cinnamomi Cassiae* (twig, Chinese herbal name: Gui-Zhi),* Radix Aconiti Lateralis Praeparata* (root, Chinese herbal name: Fu-Zi),* Radix Achyranthis Bidentatae* (root, Chinese herbal name: Niu-Xi), and* Plantaginis Semen *(seed, Chinese herbal name: Che-Qian-Zi) [9]. As per the instructions, 5 mg Jinkui Shenqi Wan (Beijing Tong Ren Tang Pharmaceutical Technology Development Inc., Dongguan, China) was dissolved in 100 mL distilled water before administration. The experimental dose for HQSXD and JKSQW in the present study was equivalent to the corresponding clinical prescription dose for a human subject weighing 60 kg.

### 2.3. Drug Administration and Sample Collections

Rats were randomly divided into three groups of 10 animals each (each group included five males and five females). Chinese medicine HQSXD solution (14 g/kg) and medicine JKSQW solution (0.520 g/kg) were administered orally every day for three days, twice a day. The blank-control group was orally administered distilled water, and they were monitored concurrently with the HQSXD-experimental groups. At the end of the experiment, sixty minutes after the last treatment, the animals were exposed to ether anesthesia; blood samples were collected by heart puncture under aseptic conditions and then centrifuged for 15 min, to obtain serum samples. Serum of HQSXD-treated rats and control serum were inactivated at 56°C in a water bath for 30 min and filtered through a 0.22 *μ*m filter membrane, termed HQSXD-S, JKSQW-control-S, and BLANK-control-S, respectively, and then stored at −80°C.

### 2.4. Primary Osteoblasts Culture and Assay for Osteoblast Proliferation

Primary rat osteoblast cells were obtained from 1-day-old neonatal Sprague-Dawley rats as described previously [10]. Primary osteoblasts were cultured by seeding 96-well plates with a density of 1 × 10^4^ per well and incubated for 24 h. After adhesion of cells, Dulbecco's Modified Eagle Medium (DMEM) was added and incubated for another 24 hours. Next, after discarding the medium, test (HQSXD-S) and control (JKSQW-control-S, BLANK-control-S) samples were added at a concentration of 10% (v/v), and the cells were incubated at 37°C in a humid atmosphere containing 5% CO_2_ for 72 h. Thereafter, 5 mg/mL of 3-(4,5-dimethylthiazol-2-yl)-2,5-diphenyltetrazolium bromide (MTT) was added and incubated for 4 hours, after which the medium was discarded, and dimethyl sulfoxide (DMSO) (150 *μ*L) was added. Absorbance was measured at 490 nm using a Synergy 2 multifunctional microplate reader (Bio-Tek) to assess osteoblast proliferation.

### 2.5. ALP Activity and Staining Assay

Osteoblasts were suspended in DMEM to obtain a cell density of 5 × 10^5^/mL; 2 mL aliquots of the cell suspension were added to 6-well plates. After 24 h of incubation, the medium was changed and cells were incubated with test (HQSXD-S) and control (JKSQW-control-S, BLANK-control-S) at a concentration of 10% (v/v) for 3 days. Alkaline phosphatase (ALP) activity was measured using an ALP assay kit (Sigma) as described previously [11]. For ALP staining, after incubation with HQSXD-S, JKSQW-control-S, and BLANK-control-S (concentration of 10% (v/v)) for three days, the cells were fixed in 70% ethanol for 15 min, washed, and then incubated with ALP staining buffer, nitro blue tetrazolium/5-bromo-4-chloro-3-indolyl phosphate (NBT/BCIP) (Beyotime Institute of Biotechnology, China), at 37°C for 30 min, air dried, and photographed.

### 2.6. Mineralization Assay

After 21 days of differentiation, the mineralization of osteoblasts was analyzed as described previously [12]. Briefly, the cells were washed with phosphate buffered saline (PBS) and fixed with 70% ethanol for one hour. The cells were then rinsed in distilled water, stained with 0.5% Alizarin Red S (ARS) at pH 4.2 with rotation for 30 min at 37°C, and subsequently washed with distilled water and dried in air. Stained cultures were photographed. To analyze ARS activity, the ARS in stained cells was destained with 10% cetylpyridinium chloride (CPC) monohydrate solution (Sigma) for 30 min with shaking. The absorbance was measured at 562 nm using a Synergy 2 multifunctional microplate reader.

### 2.7. Protein Extraction

Osteoblasts cells were incubated in 50 mL culture flasks and grown to subconfluence (approximately 60%–70%) and then treated with test (HQSXD-S) and control (JKSQW-control-S, BLANK-control-S) at a concentration of 10% (v/v) for 72 h, respectively. At the end of the incubation period, osteoblasts were collected by centrifugation at 1,000 rpm (4°C). The osteoblasts were washed twice with cold PBS, after discarding the medium. Total proteins were extracted in a chilled lysis buffer containing 7 M urea, 2 M thiourea, 4% (w/v) CHAPS, 40 mM dithiothreitol (DTT), 2% (v/v) IPG buffer, pH 3–10, 4 *μ*g/mL protease inhibitor mixture, and 4 *μ*g/mL phosphatase inhibitors. After addition of the chilled lysis buffer, the cell solution was kept oscillating for 1 h at 4°C to solubilize the proteins. The homogenate was subsequently centrifuged for 15 min at 14,000 rpm at 4°C. Total protein concentration was quantified using the Bradford assay (Amresco).

### 2.8. Two-Dimensional Electrophoresis (2DE) and Image Analysis

Electrophoresis was carried out at least thrice for reproducibility. Two-dimensional electrophoresis (2DE) was carried out as described previously [13] with some modifications. For the first-dimension isoelectric focusing gel, 250 *μ*g of protein was used for isoelectric focusing (IEF) using 17 cm nonlinear (NL) IPG strips (pH 3–10), which were immersed in rehydration buffer (8 M urea, 2% CHAPS, 25 mM DTT, 0.2% (w/v) Bio-Lyte, 0.1% bromophenol blue, and 1% IPG buffer). The strips were covered with mineral oil (GE Healthcare) to prevent samples from evaporation and rehydrated for 10–20 hours. Isoelectric focusing gel electrophoresis was performed by using an electrophoresis apparatus (GE Healthcare). The running conditions of the IEF process were as follows: 100 V, 1 h; 200 V, 1 h; 300 V, 1 h; 500 V, 1 h; 1,000 V, 1 h; 2,000 V, 1 h; and 8,000 V, up 60,000 V. After the IEF, the IPG strips were equilibrated with equilibration buffer-1 (6 M urea, 2% SDS, 20% glycerol, 0.375 M Tris-HCl [pH 8.8], bromophenol blue dye, and 1% [w/v] DTT) for 15 min and then were repeated for an additional 15 minutes in 5 mL of equilibration buffer-2, except that DTT was replaced by 2.5% [w/v] iodoacetamide. The second dimension was done with a 12.5% acrylamide gel at 10 mA/gel for one hour and then at 38 mA/gel until the bromophenol blue dye reached the bottom of the gel. After electrophoresis, the gels were visualized by silver nitrate staining. Gels were scanned using the ImageScanner (GE Healthcare). The images were analyzed with ImageMaster 2D Platinum v7.0 software (GE Healthcare, San Francisco, CA) including spot detection, background subtraction, gel matching, and normalization. Spots were detected and matched automatically to a master gel and then edited manually. Matched spots from triplicate gel sets that showed overlap ratio with an absolute value ≥2 were recognized as differentially expressed. Differentially abundant spots were selected for mass spectrometry (MS) analysis.

### 2.9. Mass Spectrometry and Database Search

Protein spots were excised from the 2DE gels using a pipette tip. Gel pieces were destained in a solution of 15 mM potassium ferricyanide and 50 mM sodium thiosulfate (1 : 1), washed with deionized water, and dehydrated in 100% acetonitrile (ACN). Samples were rehydrated for digestion with trypsin (12.5 mg/mL) at 4°C for 30 min. Excess trypsin solution was replaced with 25 mM ammonium bicarbonate. The samples were incubated overnight at 37°C. Peptides were then extracted twice with 50% ACN/5% TFA followed by 100% ACN for 15 min each. After drying, the peptide extracts were desalted with ZipTip Pipette Tips (Millipore). Mass spectrometry was done using an ultraflex III MALDI-TOF/TOF-MS (Bruker) with a high voltage of 20 kV, and spectra were externally calibrated using the peptide standard Maker. Protein identification was determined by matching the peptide mass fingerprinting (PMF) and MALDI-TOF/TOF-MS results via MASCOT (version 2.2, Matrix Science) against NCBInr database with BioTools software. Database searches were performed using the following parameters: taxonomy, rice; enzyme, trypsin; and one missed cleavage allowed. Carbamidomethylation was selected as a fixed modification, and the oxidation was allowed as a variable. PMF tolerance set to 100 ppm, and MS/MS tolerance set to 0.7 Da. A protein was regarded as identified if the MASCOT protein score was above the 5% significance threshold for the database (score >64).

### 2.10. Western Blotting Analysis

To verify the results of 2DE of the identified proteins, we randomly chose four proteins for Western blot: FPR2, TCEB1, PHB, and alpha-spectrin. Cytosolic extracts were prepared from cells, and the protein in the supernatant was quantified using the BCA protein assay kit (Beyotime Institute of Biotechnology, China). A sample (50 *μ*g) was electrophoresed on a 10% SDS-polyacrylamide gel and subsequently transferred onto a PVDF membrane (Millipore). After blocking with 5% nonfat dry milk, the membranes were incubated with anti-FPR2 (M-73, sc-66901, Santa Cruz Biotechnology), anti-alpha-spectrin (C-11, sc-46696, Santa Cruz Biotechnology), anti-TCEB1 (ProteinTech Group, Inc., China), and anti-PHB (ProteinTech Group, Inc., China). The bound antibodies were detected using a horseradish peroxidase- (HRP-) conjugated secondary antibody and visualized by an enhanced chemiluminescence detection system, followed by quantification using the Image J2x.

### 2.11. Statistical Analysis

Data are presented as mean ± SD of triplicate samples. Comparisons were performed using one-way analysis of variance (ANOVA) followed by Dunnett's test, and the difference was considered statistically significant if *p* < 0.05.

## 3. Results

### 3.1. Effects of HQSXD-Treated Rat Serum on Proliferation of Osteoblasts

**Figure 1:**
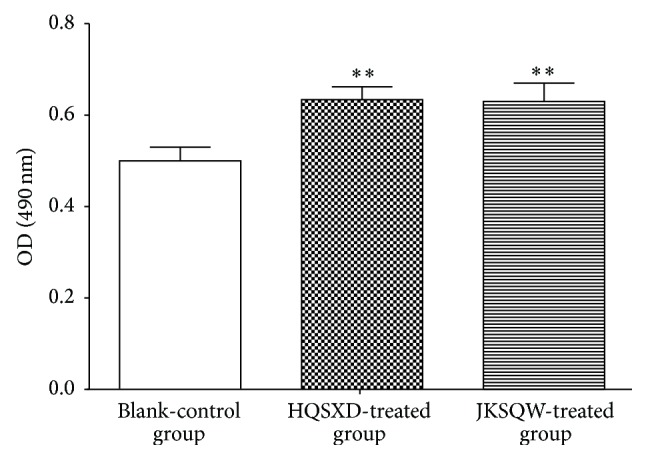
Effects of drugs on proliferation of osteoblasts. Although there was some improvement in the proliferation of osteoblasts in the HQSXD-treated group, it was not statistically significant when compared to the values observed in the group given JKSQW-control-S. Data are expressed as the mean ± SD.

The proliferation of primary osteoblasts showed an upward trend compared to that of blank control. However, there was no significant difference detected in the proliferation between HQSXD-treated and JKSQW-treated groups. Similar changes in proliferation between JKSQW-treated group and HQSXD-treated group indicated that HQSXD had an influence on osteoblasts (Table 1, Figure 1).

### 3.2. HQSXD-Treated Rat Serum Enhances Primary Osteoblast Differentiation

**Figure 2:**
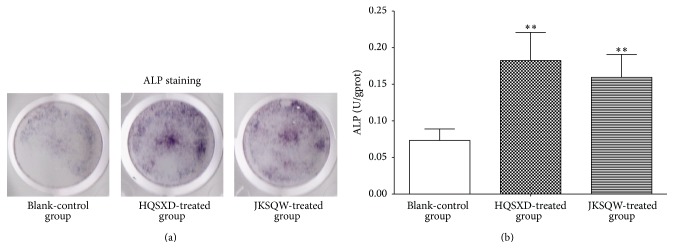
HQSXD-S enhances primary osteoblast differentiation. (a) Primary osteoblasts were treated with various drug-treated rat serums for 3 days. ALP-positive cells were stained with* ALP *solution. (b) Effect of HQSXD-S on alkaline phosphatase activity in primary culture osteoblasts. Data are means ± SD of six replicates. ^*∗∗*^ *p* < 0.01 versus control.

**Figure 3:**
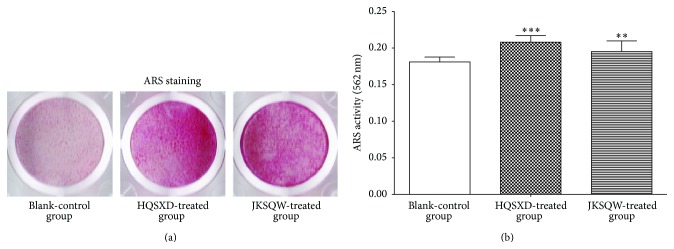
HQSXD-S promotes bone mineralization during osteoblastogenesis. (a) Calcium deposits stained with Alizarin Red solution seen in primary osteoblasts treated with various drug-treated rat serums for 21 days. (b) Stained calcium deposits were destained with 10% CPC buffer to measure the level of staining. Data are means ± SD of six replicates. ^*∗∗*^ *p* < 0.01, ^*∗∗∗*^ *p* < 0.001 versus control.

ALP is an important biochemical marker of differentiated osteoblasts, and the effects of drug on ALP activities in osteoblasts were first determined. Results of ALP staining showed that HQSXD-S and JKSQW-control-S stimulated osteoblast differentiation (Figure 2(a)). The cells cultured with HQSXD-S showed a significantly higher ALP activity than that cultured with BLANK-control-S (Figure 2(b)). ARS staining in osteoblasts was assessed after 21 days of incubation to examine whether HQSXD enhanced bone mineralization during osteoblastogenesis. In osteoblasts treated with BLANK-control-S, the calcium deposition in the mineralized matrix was minimal. The proportional areas of Alizarin Red-positive staining in the HQSXD-treated group and JKSQW-treated group were higher than that in the Blank-control group (Figure 3(a)). As shown in Figure 3(b), the level and intensity of ARS staining indicated the extent of mineralization, which increased upon treatment with HQSXD-S.

### 3.3. Protein Expression Profile in HQSXD-Treated and HQSXD-Untreated Osteoblasts

**Figure 4:**
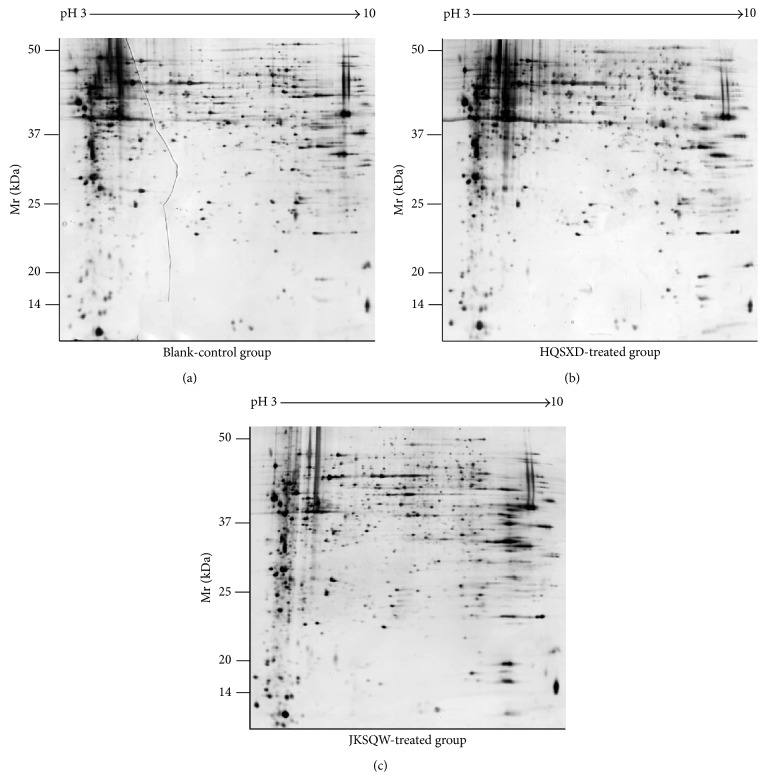
Representative silver nitrate stained gels showing two-dimensional electrophoresis protein profiles of (a) blank-control group, (b) HQSXD-treated group, and (c) JKSQW-treated group. Molecular weight (MW, kDa) and isoelectric point (*P*_*I*_) are indicated along the *y*- and *x*-axes, respectively.

**Figure 5:**
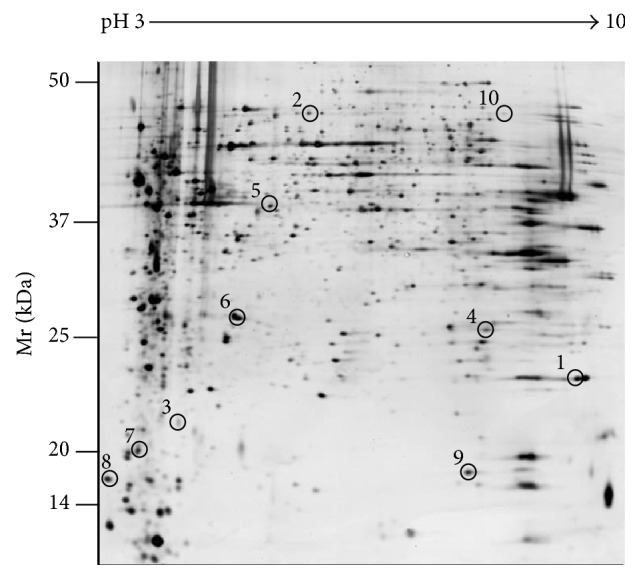
Representative 2DE gel image. Ten spots that were statistically significant (*p* < 0.05) are shown in the map and indicated by numbers. All were cut from the gels for further identification by MALDI-TOF/TOF-MS analysis, outlined in Table 2.

**Figure 6:**
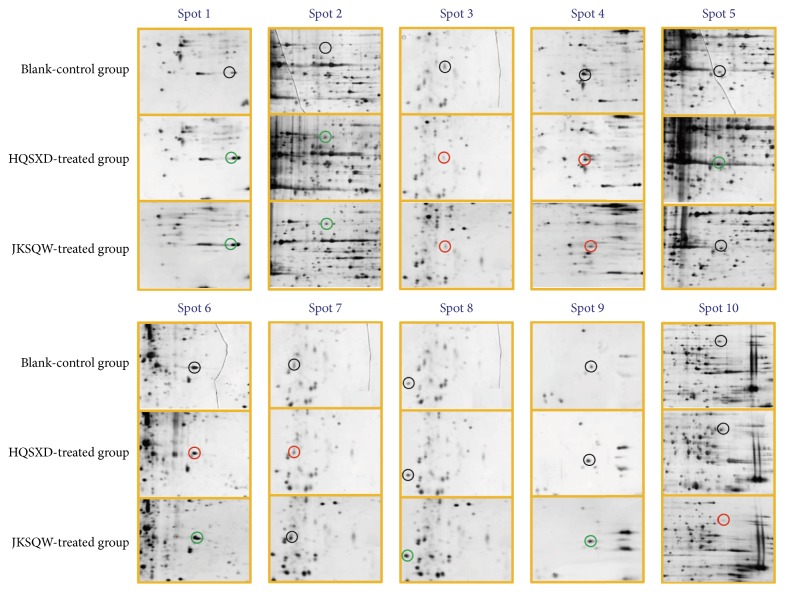
The ten protein spots of osteoblasts that were treated with and without HQSXD-S. Selected regions showed significant differences in the protein expression profile of osteoblasts among the three groups. Upregulated spots are indicated by green circles and downregulated ones by red circles.

Two-dimensional electrophoresis and gel sliver nitrate staining were conducted to further investigate the differential protein expression between HQSXD-S-treated and HQSXD-S-untreated osteoblasts. After optimization of the 2DE gels, with representative 2DE gel images shown in Figure 4, approximately 938 ± 26, 875 ± 34, and 904 ± 22 protein spots were detected in blank protein sample, HQSXD protein sample, and JKSQW protein sample, respectively. During analysis with ImageMaster 2D Platinum, spots with an overlap ratio absolute value ≥2 were recognized as differentially expressed. Thirty-eight protein spots were found to be significantly regulated among three groups, of which 15 spots were downregulated and 23 spots upregulated. Ten of these 38 spots exhibited a more than twofold increase or decrease in abundance as observed in all replicate gels. These 10 regulated proteins were indicated by the circle in Figure 5, and the selected regions that showed significant differences in protein expression profile of osteoblasts among three groups were shown in Figure 6. All of them were excised from the gels for further identification by MALDI-TOF/TOF-MS analysis.

### 3.4. Identification of the Differentially Expressed Proteins

**Figure 7:**
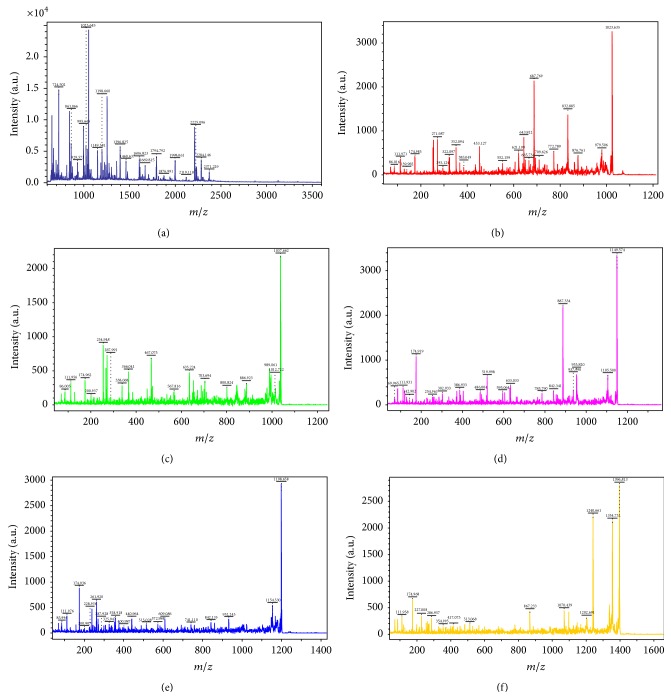
The results of the MALDI-TOF/TPF-MS analysis of protein spot 6. (a) Peptide mass fingerprinting and ((b)–(f)) peptide fragment fingerprinting of spot 6.

Proteins were identified by MALDI-TOF/TOF-MS. Ten peptide mass fingerprints (PMFs) and 50 peptide fragment fingerprints (PFF) were successfully obtained. A selected PMF of protein spot 6 is displayed in Figure 7(a), and the TOF/TOF analysis is shown in Figures 7(b)–7(f). All PMFs were evaluated with the Mascot software in NCBInr database to identify the protein spots. The result had high confidence if the protein was ranked as the best hit with a significant score and high sequence coverage. Finally, we identified eight proteins in these spots. Properties of the identification of eight selected protein spots are summarized in Table 2.

### 3.5. Effect of HQSXD on the Expression of Proteins That Regulate Antiosteoporotic Activity

**Figure 8:**
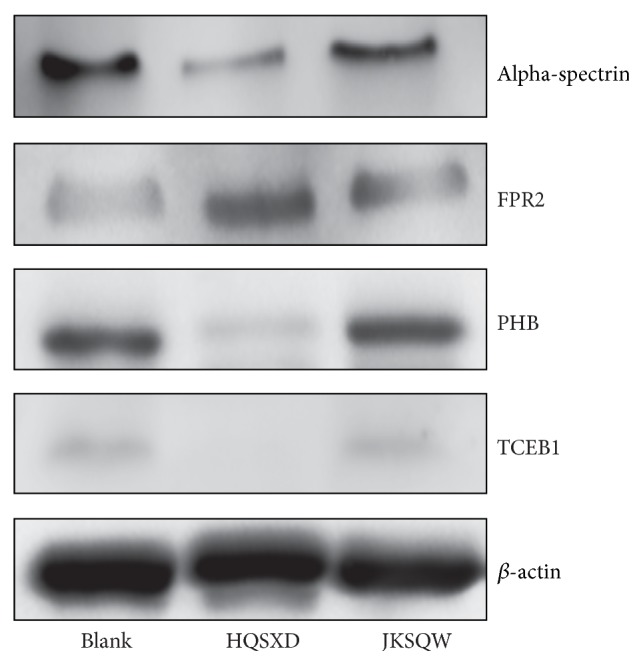
HQSXD-S treatment decreased alpha-spectrin, PHB, and TCEB1 expression and increased FPR2 expression. Cell lysates (50 *μ*g) were processed for Western blot analysis. Normalization performed to *β*-actin. The bands shown here were from a representative experiment repeated three times.

To further investigate the influence of HQSXD on the expression of antiosteoporotic proteins, we examined the expression of FPR2, alpha-spectrin, PHB, and TCEB1 by Western blotting. The expression of FPR2 was increased by treatment with HQSXD-S in comparison to the blank-control group. However, HQSXD remarkably decreased alpha-spectrin, PHB, and TCEB1 protein levels compared with blank-control group (Figure 8). Results from Western blot manifested the same trend as from proteomic analysis.

## 4. Discussion

In this study, we found that the proliferative activities of osteoblasts between HQSXD-S-treated group and JKSQW-control-S-treated group were similar. This investigation demonstrates that HQSXD can significantly facilitate bone formation through increasing the number of osteoblasts, which is beneficial to the treatment of osteoporosis. By measuring ALP activity, HQSXD was first screened for its ability to induce osteogenesis. HQSXD is capable of significantly promoting osteoblast differentiation, as well as increasing osteoblast mineralization.

Results of the identification of the selected protein spots are summarized in Table 2. The molecular weight (Mr) and isoelectric point (*P*_*I*_) of each protein spot shown in Table 2 are theoretical values. The eight protein spots were identified as (1) N-formyl peptide receptor 2 (FPR2); (2) alpha-spectrin; (3) heparan sulfate (glucosamine) 3-O-sulfotransferase 3A1 (HS3ST3A1); (4) prohibitin (PHB); (5) transcription elongation factor B (SIII), polypeptide 1 (TCEB1); (6) chromosome segregation protein; (7) nucleoside diphosphate kinase; and (8) mast cell carboxypeptidase A.We detected some proteins related to transcription, cell proliferation, differentiation, and apoptosis, such as FPR2, prohibitin, alpha-spectrin, heparan sulfate (glucosamine) 3-O-sulfotransferase 3A1, and transcription elongation factor B (SIII), polypeptide 1, which varied greatly after HQSXD treatment.

Our results suggest that HQSXD can upregulate the expression of FPR2. N-Formyl peptide receptor (FPR) is a G protein-coupled receptor, which modulates stromal cell differentiation [14] and binds to N-formyl peptides, such as N-formyl-methionyl-leucyl-phenylalanine (fMLP). Our study has shown that fMLP enhances the differentiation of MSCs into osteoblasts via an FPR-mediated signaling pathway and results in bone formation [15]. The FPR2 receptor belongs to the formyl peptide receptor family that is involved in signaling stem cell adhesion, migration, and homing for injured and inflamed tissues awaiting repair; this could potentially be exploited to direct the stem cells to target specific tissue site [16]. Therefore, the current results imply that treatment with HQSXD might promote the formation of osteoblasts by upregulating the expression level of FPR2.

We found that HQSXD could inhibit the expression of alpha-spectrin. Alpha-spectrin includes two genes encoding for alpha-I subunit and alpha-II subunit, each of them presenting its specific cellular expression pattern. Alpha-II spectrin deficiency is associated with cell proliferation defects, due to cell cycle arrest in the G1 phase (first gap phase) [17]. Spectrin and protein kinase C theta were observed in aggregates during the early stage of apoptosis [18]. Hence, we presume that alpha-spectrin is involved in the antiosteoporotic effect of HQSXD.

In this study, PHB was downregulated in HQSXD-treated osteoblasts. Prohibitin, a highly conservative protein, regulates the cohesion of sister chromatids, cellular signaling, mitochondrial biogenesis [19], cell proliferation, differentiation, apoptosis, and gene transcription [20, 21]. PHB blocks the transition of cells from G1 phase to S phase of the cell cycle, thereby arresting cell proliferation [22, 23]. PHB has been reported to affect the apoptotic pathways by repressing the transcriptional activity of E2F1 [24, 25]. As mentioned above, it is possible that HQSXD induces proliferation, differentiation, and apoptosis partly through downregulating expression of PHB in osteoblasts. It is likely that PHB might be the novel candidate in the new antiosteoporotic drug screening.

The expression of TCEB1 was downregulated in HQSXD-treatment osteoblasts. TCEB1, a 13 kDa protein also named elongin C, was originally identified as a member of the mammalian transcription factor SIII that increases the rate of transcription by suppressing RNA polymerase II pausing [26]. As part of a family of separate complexes containing elongin B and various substrate specificity factors, it acts as an E3 ubiquitin ligase [27]. Elongin B (ELB 1) and elongin C (ELC1) form a stable complex, and that depletion of either gene product by RNA-mediated interference (RNAi) causes pronounced defects in the second meiotic division and arrest of germ cell proliferation in G1 [28]. Therefore, it is possible that that TCEB1 is involved in the antiosteoporotic effect of HQSXD.

Although this study has thrown some light on the mechanism of HQSXD action, we failed to characterize the well-identified protein (e.g., HS3ST3A1) closely involved in osteoporosis. HS3ST3A1, a member of the heparan sulfate biosynthetic enzyme family, possesses heparan sulfate glucosaminyl 3-O-sulfotransferase activity. Depletion of Hs3st-A in enterocytes results in increased intestinal stem cell proliferation and tissue homeostasis loss [29].

## 5. Conclusions

The results confirm that HQSXD has a beneficial effect on osteoblasts and alters the expression level of some proteins in osteoblasts. The protein expressed by osteoblasts treated with HQSXD may be involved in cell proliferation and differentiation and other physiological processes and in the regulation of cell activation. Further study is needed to investigate the effects of major active constituents in HQSXD on protein expression on osteoblast so as to demonstrate the interaction and synergistic mechanism.

## Figures and Tables

**Table 1:** Effects of drugs on proliferation of osteoblasts.

Groups	*N*	Oral administration dose (g·kg^−1^)	Serum additive volume (%)	OD value
Blank-control	5	—	10	0.50 ± 0.03
HQSXD-treated	5	14	10	0.634 ± 0.028^*∗∗*^
JKSQW-treated	5	0.520	10	0.63 ± 0.04^*∗∗*^

Note: ^*∗∗*^ *p *< 0.01 compared with BLANK-control-S.

**Table 2:** Summary of differentially expressed proteins in osteoblasts treated with HQSXD.

Spot number^a^	Protein score^b^	Matching peptides (number)	Theoretical *P*_*I*_^c^	Theoretical Mr (Da)^c^	Target protein	Species
1	70	6	9.27	39299	N-Formyl peptide receptor 2	*Rattus norvegicus *
3	67	7	5.65	54851	Alpha-spectrin	*Rattus norvegicus *
5	76	6	10.08	43712	Heparan sulfate (glucosamine) 3-O-sulfotransferase 3A1	*Rattus norvegicus *
6	173	12	5.57	29859	Prohibitin	*Rattus norvegicus *
7	67	4	4.59	12752	Transcription elongation factor B (SIII), polypeptide 1	*Rattus norvegicus *
8	70	11	4.98	127444	Chromosome segregation protein	*Rattus norvegicus *
9	101	4	6.91	17386	Nucleoside diphosphate kinase	*Rattus norvegicus *
10	84	12	8.86	48199	Mast cell carboxypeptidase A	*Rattus norvegicus *

^a^Protein spot number according to Figure 3.

^b^Protein scores were based on combined mass and mass/mass spectra from MALDI-TOF/TOF identification MS.

^c^Theoretical molecular mass (Mr) and isoelectric point (*P*_*I*_) from the NCBInr database.
